# Biochar granulation reduces substrate erosion on green roofs

**DOI:** 10.1007/s42773-022-00186-7

**Published:** 2022-10-27

**Authors:** Wenxi Liao, Melanie A. Sifton, Sean C. Thomas

**Affiliations:** Institute of Forestry and Conservation, John H Daniels Faculty of Architecture Landscape and Design, University of Toronto, 33 Willcocks St., Toronto, ON M5S 3B3 Canada

**Keywords:** Charcoal, Post-processing, Dust suppressant, Erosion control, Vegetation, Green infrastructure

## Abstract

Green roofs are exposed to high winds and harsh environmental conditions that can degrade vegetation and erode substrate material, with negative consequences to ecosystem services. Biochar has been promoted as an effective substrate additive to enhance plant performance, but unprocessed biochars are susceptible to wind and water erosion. Applications of granulated biochars or chemical dust suppressants are suggested as a means to mitigate biochar and substrate erosion; however, research on biochar type and chemical dust suppressant use on biochar and substrate erosion is lacking. Vegetation is a crucial factor that influences substrate erosion, yet plant responses may vary with biochar type and chemical dust suppressant; thus, the effects of possible mitigation measures on biochar and substrate erosion are unclear. We investigated the effects of surface-applied granulated and unprocessed biochars and an organic dust suppressant (Entac™) on biochar and substrate erosion on green roofs with *Sedum album* L. and a native plant mix. Our results show that 94% of unprocessed biochars were lost from green roofs after 2 years regardless of the Entac™ amendment, likely due to the lightweight nature and fragmentation of biochar particles. In contrast, granulation of biochars reduced the biochar erosion and total substrate erosion by 74% and 39%, respectively, possibly due to enhanced biochar bulk density and particle size and improved moisture retention of biochar-amended substrates. Additionally, *Sedum album* better reduced biochar and substrate erosion than the native plant mix, likely due to rapid development of high vegetation cover that reduced wind exposure and enhanced substrate moisture retention. We conclude that applications of granulated biochars can substantially reduce biochar and substrate erosion on green roofs, improving green roof sustainability.

**Graphical Abstract:**

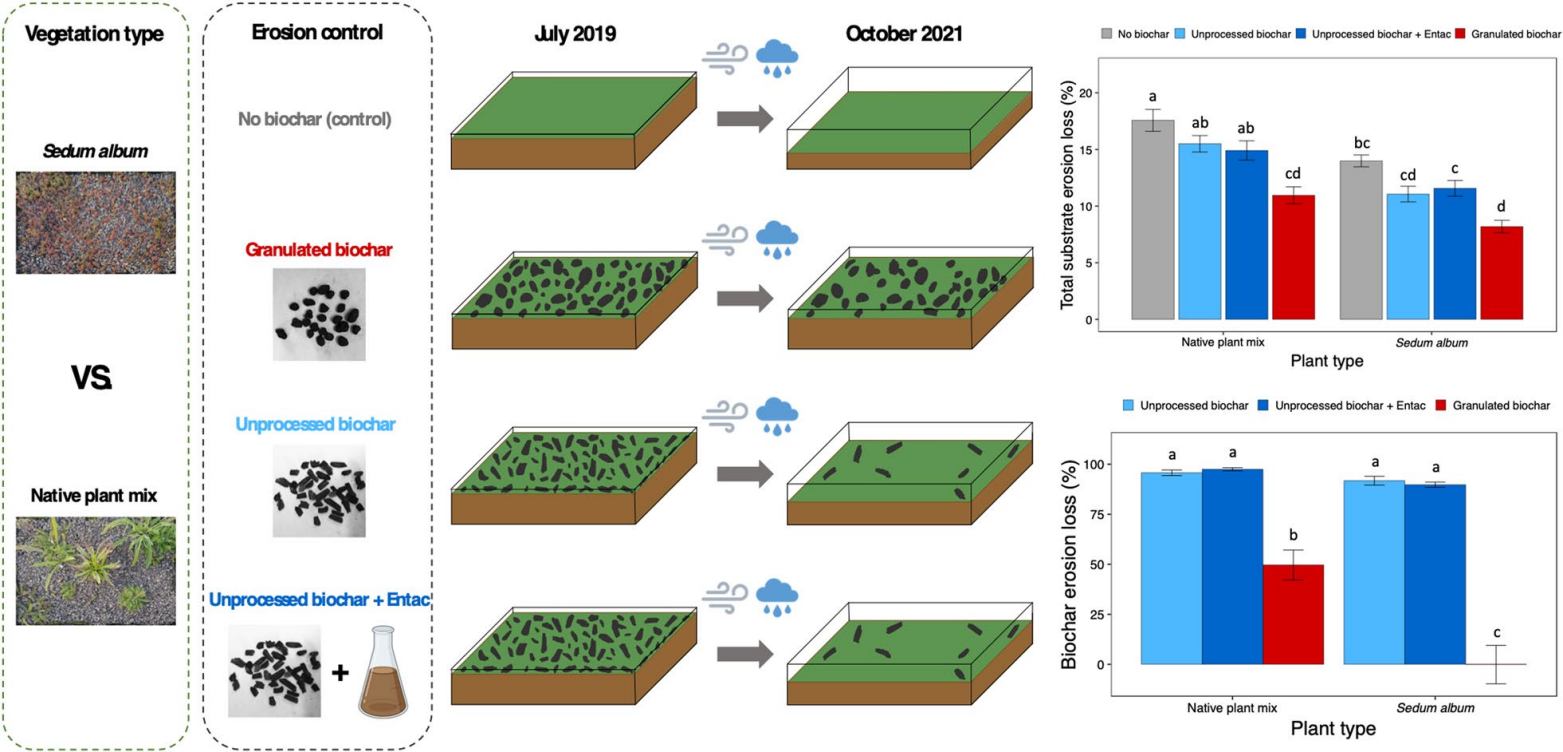

**Supplementary Information:**

The online version contains supplementary material available at 10.1007/s42773-022-00186-7.

## Highlights

- Biochar granulation substantially reduces biochar erosion and total substrate erosion.
- Although biochar reduces substrate erosion loss, almost all the unprocessed biochars were lost due to erosion after 2 years.
- *Sedum album* better mitigates biochar erosion and total substrate erosion than native plant mix.

**Supplementary Information:**

The online version contains supplementary material available at 10.1007/s42773-022-00186-7.

## Introduction

Green roofs, which are roofs covered with plants rooted in engineered substrates, have been increasingly promoted worldwide through policies that aim to alleviate environmental problems and enhance ecosystem services in cities (Li and Yeung 2014; Shafique et al. 2018). However, vegetation degradation is commonly observed on green roofs due to harsh growth conditions, such as severe drought stress (Durhman et al. 2007; Rowe et al. 2012); loss of vegetation cover reduces ecological benefits provided by roof systems. As green roofs are often exposed to very high wind and intense rain events (Oberndorfer et al. 2007), low vegetation cover is also expected to result in substrate erosion losses (Cascone 2019). Such erosion losses would in turn reduce the capacity of the green roof system to support vegetation and may directly contribute to environmental pollution.

Biochar, which is a porous carbon-rich material produced by pyrolyzing biomass, has been suggested as an effective substrate additive on green roofs. Biochars generally show a high water-retention capacity (Razzaghi et al. 2020), and the capacity to sorb and retain plant nutrients (Laird and Rogovska 2015). Studies have shown that biochar applications improve plant performance on green roofs by enhancing substrate moisture content and plant nutrient availability (Chen et al. 2018a; Liao et al. 2022a). Biochar amendments to green roof substrates may provide additional benefits, including improved stormwater management (Kuoppamäki et al. 2016), increased carbon sequestration (Chen et al. 2018b), decreased decomposition (Spokas 2010), and improved discharge water quality (Beck et al. 2011; Liao et al. 2022b).

Biochars generally have a low bulk density (Chia et al. 2015); this can provide a benefit to green roof systems by reducing system structural load (Cao et al. 2014) but is likely to contribute to susceptibility to wind and water erosion (Blanco-Canqui 2019; Gelardi et al. 2019). Wind tunnel studies have suggested that biochar addition increases particulate matter emissions (e.g., PM_10_ and PM_100_) due to presence of fine biochar particles (Ravi et al. 2016), abrasion and fragmentation of larger biochar particles (Ravi et al. 2016), and mutual repulsion among biochar and soil particles (Li et al. 2018). Water transport can also be significant (Rumpel et al. 2015), with small-scale experiments reporting 7–55% losses of surface-deposited pyrogenic carbon in a single intense rainstorm (Rumpel et al. 2009). Addition of conventional biochar (i.e., not subject to post-processing treatments such as oxidation or granulation; hereinafter “unprocessed biochar”) to green roof substrates has been found to increase total suspended solids in discharge water due to biochar fragmentation (Liao et al. 2022b). Biochar usually absorbs heavy metals and organic contaminants in soils due to its large surface area and high sorption capacity (Nartey and Zhao 2014; Gelardi et al. 2019). Therefore, as biochars are eroded, biochar-bound pollutants may be released and suspended in the atmosphere (Gelardi et al. 2019) and/or deposited as sediments and become bioavailable in watersheds (Rumpel et al. 2015), which could eventually pose threats to ecosystems and to human health (Rumpel et al. 2015; Gelardi et al. 2019).

The use of granulated or pelletized biochars (Thomas 2021) or chemical dust suppressants (Jin et al. 2019; Wang et al. 2021) has been suggested as a means to mitigate biochar and substrate erosion. Granulated biochars (formed by a granulation process with a binding agent) are expected to be resistant to wind and water erosion because they have higher bulk density and lower total porosity compared with unprocessed biochars (Liao et al. 2022a). Granulated biochars are approximately spherical in shape, and show increased crushing strength relative to unmodified biochars (Briens and Bowden-Green 2019, 2020); thus, biochar granules may not be readily fragmented into fine particles. Biochar amendment can also influence total erosion of biochar and substrate particles, but the effects may vary with biochar type. Biochar improves substrate stability by increasing substrate water content (Li et al. 2018) and enhancing substrate aggregation (e.g., by forming water-stable aggregates) (Blanco-Canqui 2017). In a recent study we found that applications of unprocessed biochars generally better enhanced substrate water retention capacity compared to granulated biochars (Liao et al. 2022a) and may thus show increased substrate stability. In contrast, granulated biochars may reduce substrate inter-pores and impede the movement of biochar and substrate particles through the channels due to  their spherical shape (Liao and Thomas 2019). Enhanced plant performance has been reported in response to granulated biochars compared to unmodified biochars on green roof substrates (Liao et al. 2022a). This effect may also help alleviate biochar and substrate total erosion by reducing surface wind speed (Hong et al. 2020) and intercepting rainwater that could result in splash erosion (Durán Zuazo and Rodríguez Pleguezuelo 2008). Nevertheless, plant responses may vary with biochar post-processing (Thomas 2021) and the effects may also be species-specific (Liao and Thomas 2019).

A main alternative to use of granulated biochars is the use of environmentally-friendly chemical dust control agents. Studies have shown that “green” chemical dust suppressants can reduce over 90% of dust emissions from road surfaces (Wang et al. 2021) and coal (Jin et al. 2019) by binding particles and forming hardened surfaces with high mechanical strength (Kuttner and Thomas 2017; Jin et al. 2019) and increasing substrate water retention (Wang et al. 2021). Application of an organic dust suppressant with biochar was also found to enhance survivorship and growth of willow cuttings (Kuttner and Thomas 2017). However, the effects of biochar type and chemical dust suppressant on biochar erosion and total substrate erosion have been little studied, and no existing research on this topic has examined green roofs.

Vegetation cover and type are also likely to affect erosion of green roof substrates. In general, particle erosion decreases as vegetation cover increases (Zhou et al. 2006; Durán Zuazo and Rodríguez Pleguezuelo 2008). Plant roots help stabilize substrates by enhancing substrate aggregation, increasing bonding strength between roots and particles, and improving substrate structure (Gyssels et al. 2005). Moreover, plant aboveground biomass absorbs airflow momentum and thus reduces wind speed at substrate surfaces (Hong et al. 2020); plants also intercept rainwater, reducing splash erosion and surface flow (Durán Zuazo and Rodríguez Pleguezuelo 2008). Vegetation type can affect substrate erosion. Different plant species have distinct root systems and root-substrate adhesion and thus are associated with differences in substrate physical consolidation and substrate aggregate stability (Fattet et al. 2011; Luo et al. 2020). In addition, plant structure, such as leaf shape, height, and surface features such as hairs, may influence particle wind and water erosion due to differences in surface roughness (Luo et al. 2020); plant species likewise vary in terms of canopy rainwater interception, transpiration, and water repellency (Cerdà et al. 2021). *Sedum* species, which are commonly planted on green roofs, form vegetation mats that can reduce substrate erosion loss through substrate reinforcement (Andry et al. 2007). Erect perennial herbs and sub-shrubs, being increasingly used on green roofs due to additional ecological benefits (Dvorak and Volder 2010), may also mitigate particle erosion as the aboveground biomass intercepts raindrops and reduces surface flow (Zhao et al. 2019). However, essentially no research has been done on the effects of vegetation type and coverage on erosion control on green roofs.

In the present study, we investigated the effects of biochar type and organic dust suppressant use on biochar and substrate erosion on green roofs with two distinct vegetation types. The following hypotheses were tested: (1) biochar granulation and use of organic dust suppressants will reduce biochar erosion and total substrate erosion; (2) granulated biochar will better reduce biochar and substrate total erosion than unprocessed biochar due to reduced substrate porosity and enhanced plant performance; (3) *Sedum album* and a native plant mix will show distinct effects on biochar erosion and total substrate erosion; (4) biochar and substrate erosion will decrease with increasing vegetation cover.

## Materials and methods

### Study site and climate

The experiment was performed on the Green Roof Infrastructure Small-Scale Testing Lab (GRISSTL) of the Institute of Forestry and Conservation at the University of Toronto St. George Campus (43° 39′ 40″ N, 79° 24′ 1″ W). Toronto has a temperate continental climate, with an average temperature of 10 °C and total precipitation of 831 mm based on 1981 to 2010 climate normals (Environment and Climate Change Canada 2022). Daily climate data during the experimental period (July 2019–October 2021) were retrieved from the Toronto city center station (Environment and Climate Change Canada), which is ~ 3 km from the study site. The total precipitation and mean speed of maximum wind gust during the experimental period were 1737 mm (772 mm/year) and 46 km/h (Additional file 1: Fig. S1), respectively, with prevailing winds from the west.

### Substrate, biochar, and organic dust suppressant

The substrate used in this study was a commercial extensive green roof mix (provided by Gro-Bark, Inc., Canada) that follows FLL (Forschungsgesellschaft Lanschaftsentwicklung Landschaftsbau) guidelines. The substrate was composed of 70% porous aggregates, 25% organic matter, and 5% of fine sand. Unprocessed and granulated biochars were used in the experiment. The unprocessed biochar was produced by slow pyrolysis of conifer sawmill waste (Titan Clean Energy Projects Co., Canada). The granulated biochar was made from a similar ground conifer biochar and a proprietary binder using a drum granulation process. Unprocessed and granulated biochars in 2.8–6.3 mm particle size range were prepared by dry sieving. Unprocessed biochars were heat-treated at 100 °C for 24 h to outgas potential toxic compounds present in the biochars prior to the biochar application (Gale et al., 2016). Details of properties and test methods for the substrate and the unprocessed and granulated biochars are reported in Liao et al. (2022a).

The organic dust suppressant product Entac™ (Enssolutions Group Inc., Beamsville, ON, Canada) was used in the study to mitigate biochar and substrate erosion. Entac™ is a liquid tall oil pitch emulsion that is derived from sulfate pulping of pine. It is suggested to be an effective product for dust control and surface stabilization through particle binding on surfaces and is deemed to be environmentally friendly (Preston et al. 2020). Additional characteristics of Entac™ are documented in Kuttner and Thomas (2017).

### Experimental design

A factorial experiment using green roof modules (dimension: 0.4 m in width, 0.6 m in length, and 0.09 m in depth) was carried out from July 2019 to October 2021. Two factors were included in the experiment: the first factor was erosion control treatment (granulated biochars, unprocessed biochars, unprocessed biochar and Entac™ (hereafter: BCEntac), and a control with no biochar); the second factor was vegetation type (*Sedum album* and a native plant mix). Seven replicates were implemented for each treatment and control. In total, 56 green roof modules were used in the experiment: (3 erosion control treatments + 1 control) × 2 vegetation types × 7 replicates = 56 modules. One replicate from each treatment and control was placed on an elevated table at 1 m height and these replicates were treated as blocks,   and the placement of treatment modules was randomized within blocks.

Unprocessed and granulated biochars were applied as a topdressing at a dosage of 20 t/ha (480 g/module) based on the surface area of the green roof modules (0.24 m^2^). Entac™ was diluted at 3:1 v/v deionized water to Entac ratio (Kuttner and Thomas 2017). The diluted Entac™ emulsion was sprayed to the surface of green roof modules at a rate of 568 L/ha, which is equivalent to 15 mL Entac™ emulsion per module. Each green roof module was filled up to 0.08 m with substrates or biochar-amended substrates.

Two types of vegetation were planted on the green roof modules. The native plant mix modules were planted through direct seeding of equal amounts of eight plant species native to the study region (Southern Ontario, Canada, with many also native to Eastern and Central North America; Additional file 1: Table S1). All the native plant seeds, except for *Dalea purpurea* Vent., were treated with 50 mL of a slurry of Nutri Boost ™ 1 (Nutrilife Plant Products, Canada) mixed with a microcellulose-water mixture at 0.2% v/v ratio to help break seed dormancy; the mixture of slurry and seeds was applied to each native plant mix module using 25 mL pipettes. *Dalea purpurea* was directly seeded to each native plant mix module the day after the slurry seed application. For each *Sedum album* module, 20 *Sedum album* cuttings, each with the length of 4–6 cm, were planted. The same amount of the slurry was also added to each *Sedum album* module. All the green roof modules were misted similarly with irrigation water after seeding and planting. For the first month, each module was gently irrigated with similar amount of water every two to 3 days to maintain the module at approximate field capacity. As most of the native seeds did not survive the first year establishment period due to drought stress, five similar-sized seedlings from each of three pre-grown native plant species (Additional file 1: Table S1) were transplanted to each native plant mix module in random locations in September 2020.

### Erosion and vegetation assessments

At the end of the experiment (October 2021) two substrate samples were taken from each green roof module at random locations using 123-mL stainless-steel cylinders (radius: 2.4 cm, height: 6.8 cm). The cylinders were inserted until reaching the bottom of the modules. The distance between the cylinder top and the substrate surface was measured twice at opposite positions using a ruler to a precision of 1 mm. The mean of the two distance measurements was determined to represent the cylinder top-to-substrate surface depth. The substrate thickness was calculated as the difference between the cylinder height and the cylinder top-to-substrate surface depth. The substrate sample from each cylinder was bagged individually and sample dry mass was measured using an analytical balance after drying at 65 °C for 72 h in a forced-air oven. Substrate bulk density (g/cm^3^) was calculated as: M_s_/(T_s_ × R_c_^2^ × π), where M_s_ is the dry mass of the substrate sample, T_s_ is the substrate thickness, and R_c_ is the radius of the cylinder (2.4 cm).

One of the dry substrate samples from each biochar and BCEntac module was used to assess biochar erosion loss. The substrate samples were sieved using stacked 2-mm and 1-mm sieves and collected in a sieve pan. The biochar particles retained on the 2-mm and 1-mm sieves were manually separated from other substrate particles. The mass of the biochar particles in each substrate sample was determined using an analytical balance. Biochar erosion loss was calculated as: (M_ba_ − M_bc_)/M_ba_ × 100%, where M_ba_ and M_bc_ represent the dry mass of biochars in the sampling area at the initial biochar application and at the final sample collection, respectively. The biochar dry mass at the initial application was calculated as 3.62 g per sampling area, which was determined as the cylinder surface area multiplying by the biochar application rate.

Substrate erosion was assessed by measuring substrate depth using the systematic sampling method. A coordinate grid was built for each green roof module, with southwest corner being considered as origin (0, 0) and the length and width of the module as x-axis (0–60 cm) and y-axis (0–40 cm), respectively. The substrate depth was measured from (5, 5) to (55, 35) along the x-axis and y-axis with an interval of 10 cm, yielding a total of 24 substrate depth records for each module (Additional file 1: Fig. S2). The substrate depth was determined with a 1-mm precision ruler. The substrate erosion depth at each sampling point was calculated as the difference between the initial and final substrate depth over the 2-year experiment. The percent total substrate erosion loss was determined as: D_e_/D_i_ × 100%, where D_e_ is the substrate erosion depth and D_i_ is the initial substrate depth. Substrate moisture content at ~ 1 cm from bottom of the module was determined at the same locations as substrate depth measurements using a SM150 Soil Moisture Kit (Delta-T Devices Ltd, UK). The substrate depth, erosion, and moisture measurements were conducted on three randomly selected replicates for each treatment and control. Mean substrate depth, erosion loss, and moisture content were calculated for each module. The substrate erosion loss in the modules was interpolated using an inverse distance weighting (IDW) method to estimate the spatial distribution patterns of substrate erosion (Lu and Wong 2008). Edge-to-center difference, which was calculated as the difference between the mean substrate depth along the edge and that at the center (non-edge) of the module, was used to assess the substrate loss pattern.

To investigate effects of vegetation cover on biochar and substrate erosion, plant cover was assessed using image segmentation in October 2019 (3 months after initial vegetation seeding/planting). Photos with the resolution of 12 megapixels were taken for the green roof modules using a GoPro Hero 5 Black camera (GoPro Inc., USA) on an overcast day to reduce shadow effects. The images were processed to the approximate vertical downward angle and cropped along the module boundaries. Image segmentation was performed using Otsu’s method (Otsu 1979), with two different sets of thresholds being used for the native plant mix and *Sedum album* modules due to the distinct plant colors. The accuracy of the image segmentation was assessed by comparing the ground truth and classified images and creating a basic 2 × 2 confusion matrix (quantifying correctly and incorrectly classified points) based on 150 random points of each vegetation type. The overall accuracies of image segmentation (Congalton 1991) for the native plant mix and *Sedum album* modules were 93.4% and 87.5%, respectively.

### Statistical analysis

Biochar and substrate erosion and substrate properties were analyzed using two-way analysis of variance (ANOVA) after data normality and homoscedasticity were confirmed. Post-hoc Tukey’s honestly significant difference test was conducted on variables with significant ANOVA results. The effects of substrate moisture content on substrate erosion and vegetation cover effects on biochar and substrate erosion were assessed using linear regression models, with the model assumptions verified with diagnostic plots. Results with *p* < 0.05 were considered statistically significant for all analyses. Image segmentation for vegetation cover assessments was performed using OpenCV 4.5.4 (Bradski 2000) in Python (version 3.8.8). The sf and raster packages in R (version 4.0.2) were used for spatial interpolation of substrate erosion distribution patterns. All statistical analyses were conducted in R (version 4.0.2) (R Core Team 2020).

## Results

### Biochar erosion

**Fig. 1:**
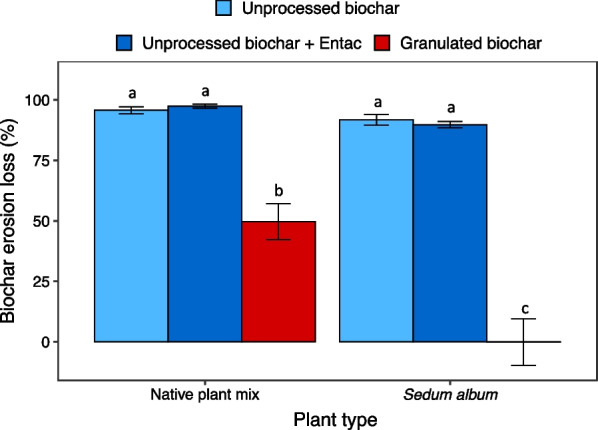
Biochar erosion loss on green roof modules. Mean biochar erosion loss in each treatment is plotted ± 1 standard error (n = 7 replicates per treatment). Bars with different lowercase letters differ significantly at *P* < 0.05 according to Tukey’s HSD test following two-way ANOVA

Biochar granulation significantly reduced biochar erosion losses, while the applications of the Entac™ to the unprocessed biochars showed no detectable effect (Fig. 1). Approximately 94% of the unprocessed biochars and BCEntac were lost due to wind and water erosion after 2 years. In contrast, the granulated biochars showed 74% lower biochar erosion loss relative to the unprocessed biochars, with only 25% of granulated biochars being lost due to erosion. In addition, the green roof modules planted with the *Sedum album* decreased biochar erosion (Table 1) compared with those planted with the native plant mix (Fig. 1). On average, 60% and 81% of biochars applied to the *Sedum album* and native plant mix modules, respectively, were lost due to erosion. Biochar treatments and vegetation also showed strong interactive effects on biochar erosion loss (*p* < 0.001: Table 1), indicating that treatment effects differed between the *Sedum album* and native plant mix modules. Nearly half of the granulated biochar applied to the native plant mix modules was lost, while all the granular biochar remained when amended to the *Sedum album* modules.

**Table 1:** Two-way ANOVA outputs for erosion and substrate property responses to biochar and vegetation type treatments

Source of variation	df	Sums of squares	Mean Square	F	*P*
Biochar erosion loss					
Biochar	2	44,362	22,181	120.9	< 0.001***
Vegetation type	1	4403	4403	24	< 0.001***
Biochar × vegetation type	2	4532	2266	12.3	< 0.001***
Residuals	36	6607	184		
Total substrate erosion loss					
Biochar	3	2822	941	24.48	< 0.001***
Vegetation type	1	1797	1797	46.78	< 0.001***
Biochar × vegetation type	3	53	18	0.46	0.71
Residuals	568	21,826	38		
Substrate depth					
Biochar	3	1806	602	24.48	< 0.001***
Vegetation type	1	1150	1150	46.78	< 0.001***
Biochar × vegetation type	3	34	11	0.46	0.71
Residuals	568	13,969	25		
Substrate moisture content					
Biochar	3	857	286	13.62	< 0.001***
Vegetation type	1	2960	2960	141.08	< 0.001***
Biochar × vegetation type	3	239	80	3.79	0.01*
Residuals	568	11,918	21		
Substrate bulk density					
Biochar	3	0.224	0.075	3.31	0.023*
Vegetation type	1	0.673	0.673	29.88	< 0.001***
Biochar × vegetation type	3	0.062	0.021	0.91	0.437
Residuals	104	2.344	0.023		
Edge-to-center difference					
Biochar	3	18.5	6.18	1.88	0.174
Vegetation type	1	15.4	15.44	4.69	0.046*
Biochar × vegetation type	3	0.3	0.11	0.03	0.991
Residuals	16	52.6	3.29		

Asterisks indicate significance of two-way ANOVA: *P < 0.05; **P < 0.01; ***P < 0.001

Statistically significant relationships (*P* < 0.05) are in boldface type

### Total substrate erosion

#### Biochar and vegetation effects

**Fig. 2:**
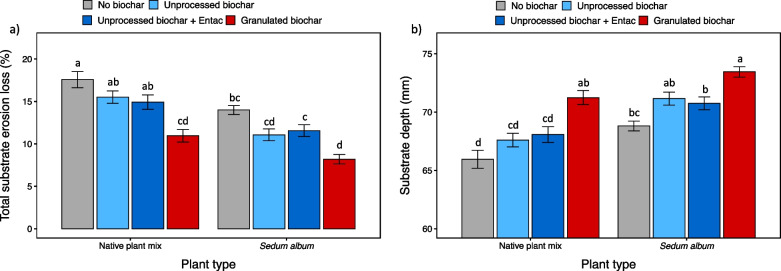
Total substrate erosion loss (**a**) and substrate depth (**b**) on green roof modules by treatment. Bars plot mean values ± 1 standard error (n = 7 replicates per treatment). Bars with different lowercase letters within each figure differ significantly at *P* < 0.05 according to Tukey’s HSD test following two-way ANOVA

Biochar addition reduced total substrate erosion (Fig. 2a), with an average reduction of 24% relative to controls. Application of granulated biochar was most effective, reducing substrate erosion loss by 39%, and showing the least total substrate erosion loss (9%) among all treatments (Fig. 2a). Additions of unprocessed biochars and BCEntac also reduced total substrate erosion compared to controls; however, no differences were observed between the unprocessed biochars with and without Entac™ (*p* > 0.05). Approximately 16% of the green roof substrate material without biochars was lost due to erosion over 2 years. Vegetation effects on erosion losses were also pronounced (Table 1): overall, the modules with *Sedum album* significantly reduced total substrate erosion compared to the native plant mix (Fig. 2a), with the *Sedum album* modules showing 24% lower total substrate erosion.

Similar trends were found for the substrate depth at the end of the experiment (Table 1): the green roof modules with biochar addition had deeper substrates than the control, with the modules amended with the granulated biochars showing the deepest substrates (72 mm) (Fig. 2b). The modules planted with *Sedum album* (71 mm) were about 4% thicker than those with the native plant mix (68 mm) (*p* < 0.001). No differences in the substrate depth were observed between the modules with the unprocessed biochar and BCEntac treatments.

#### Substrate property effects

**Fig. 3:**
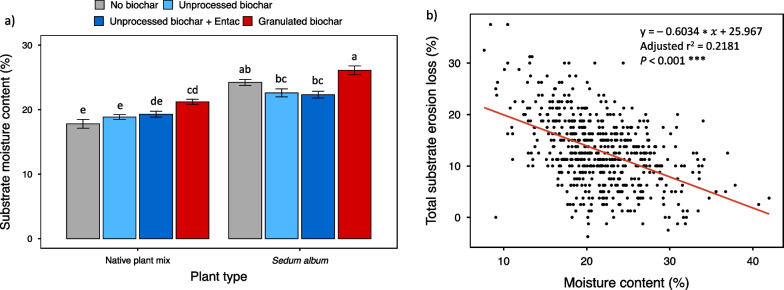
Substrate moisture content by treatment (**a**), and pooled relationship between substrate moisture content and total substrate erosion loss (**b**) on green roof modules. Bars plot mean substrate moisture content ± 1 standard error (n = 7 replicates per treatment). Bars with different lowercase letters within each figure differ significantly at *P* < 0.05 according to Tukey’s HSD test following two-way ANOVA. The fitted linear regression model pools all measurements (n = 576). Asterisks indicate significance of model: ***P < 0.001

Based on measurements at the end of the experiment, substrates amended with granulated biochars had the highest moisture content (24%) among all treatments, while the unprocessed biochar and BCEntac treatments showed no effect on the substrate moisture content (Fig. 3a). In addition, substrates with *Sedum album* had 24% higher moisture content than those with the native plant mix (*p* < 0.001). Biochar treatments and vegetation showed interactive effects on green roof substrate moisture content (*p* = 0.01: Table 1), indicating that treatment effects on substrate moisture content differed between two vegetation types. The substrate moisture content was negatively related to the total substrate erosion on green roofs (*p* < 0.001, r^2^ = 0.2195: Fig. 3b).

**Fig. 4:**
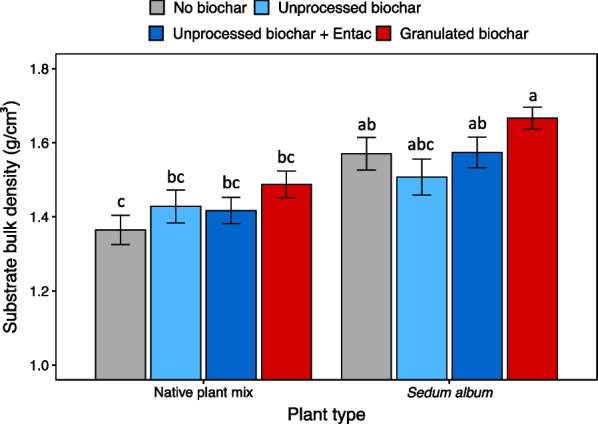
Substrate bulk density by treatment in green roof modules. Bars plot mean substrate bulk density ± 1 standard error (n = 7 replicates per treatment). Bars with different lowercase letters differ significantly at *P* < 0.05 according to Tukey’s HSD test following two-way ANOVA

The applications of the granulated biochars also resulted in higher substrate bulk density (1.58 g/cm^3^) compared to the unprocessed biochar addition treatments (1.48 g/cm^3^) and to the control (1.47 g/cm^3^) (Fig. 4). Additionally, the substrates from the *Sedum album* modules showed 11% higher bulk density than those from the native plant mix modules (Fig. 4 and Table 1).

#### Spatial distribution patterns of substrate erosion

**Fig. 5:**
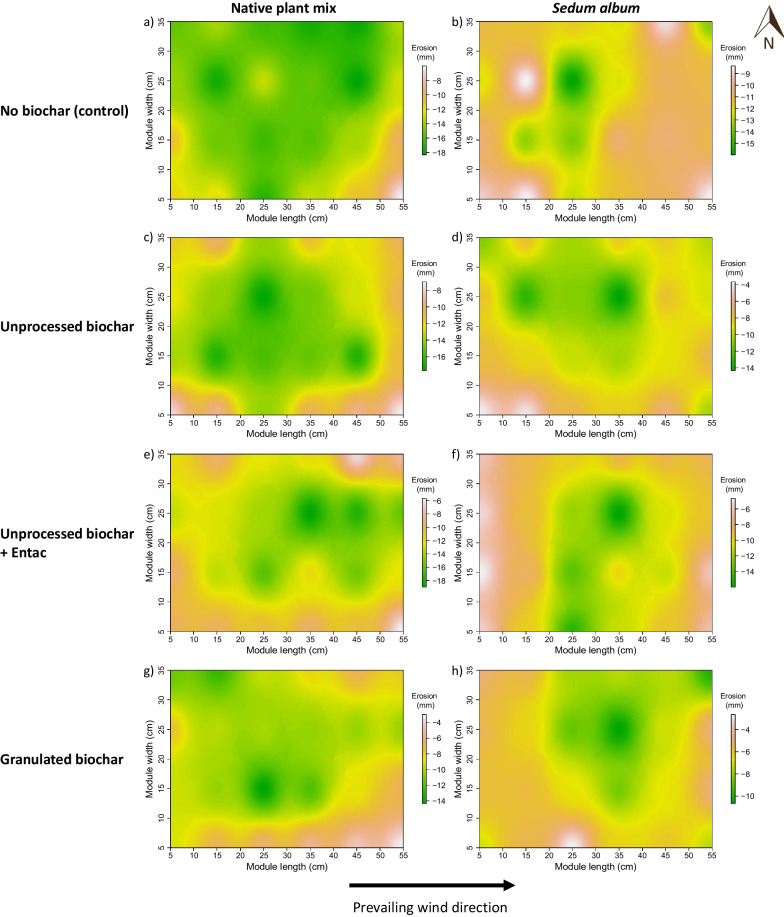
IDW spatial interpolation of mean substrate erosion in green roof modules with the native plant mix (left column) and the *Sedum album* (right column) for the control (**a**, **b**), unprocessed biochars (**c**, **d**), unprocessed biochars with Entac™ (**e**, **f**), and granulated biochars (**g**, **h**). Negative values indicate substrate erosion. Substrate erosion is based on measurements on 3 replicates for each biochar treatment and the control. White and green colors indicate low and high substrate erosion, respectively. The black arrow indicates the prevailing wind direction

Total substrate erosion losses were most pronounced near the center of the green roof modules, with less erosion along the edges (Fig. 5). The substrates in the modules with no biochar amendment generally showed a more even erosion spatial distribution, particularly the modules with the native plant mix (Fig. 5a). In contrast, the substrates with the unprocessed biochar (Fig. 5c, d) and BCEntac (Fig. 5e, f) addition had higher substrate erosion around the center than the edges of the modules. In addition, the modules with the native plant mix showed larger edge-to-center differences than those with the *Sedum album* (Additional file 1: Fig. S3 and Table 1).

### Vegetation cover effects on biochar and total substrate erosion

**Fig. 6:**
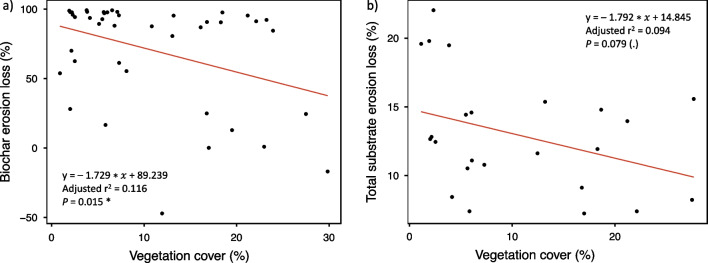
Relationship between vegetation cover in October 2019 and biochar erosion (**a**) and total substrate erosion (**b**). Lines indicate fitted linear regression models (n = 42 for biochar erosion and n = 24 for substrate erosion). Asterisks indicate significance of models: (.), *P* < 0.1; *P < 0.05

Vegetation cover was significantly related to biochar erosion (*p* = 0.015, r^2^ = 0.138), with biochar erosion decreasing with vegetation cover (Fig. 6a). Likewise, vegetation cover showed a marginally significant relationship with total substrate erosion (*p* = 0.079, r^2^ = 0.134), with total substrate erosion decreasing as vegetation cover increased (Fig. 6b).

## Discussion

As the first study to investigate biochar in relation to substrate erosion on green roofs, we found that although biochar addition reduced the total substrate erosion, almost all the unprocessed biochar added was lost due to wind and water erosion after 2 years. In contrast, additions of granulated biochars strongly reduced both biochar and total substrate erosion. Contrary to our hypothesis, the applications of the organic dust suppressant Entac™ to the unprocessed biochars showed no effect on biochar or total substrate erosion. In addition, the green roof modules with *Sedum album* showed reduced biochar and total substrate erosion compared to those with a native plant mix.

### Biochar granulation strongly reduces biochar erosion

Consistent with our initial hypothesis, granulation strongly reduced biochar erosion losses regardless of vegetation type. This was almost certainly due to higher bulk density and aggregate particle size of the granulated biochars relative to unprocessed biochars (Liao et al. 2022a), which resulted in a higher stability of the granular biochars. The spherical form of biochar granules likely also contributes to aerodynamic stability that reduces wind erosion. In addition, biochar granulation generally increases biochar crushing strength (Briens and Bowden-Green 2019, 2020); thus, the granulated biochars were less susceptible to biochar fragmentation during application and later exposure to weathering and plant root development. This finding is consistent with a recent study that reported lower leachate suspended solids with the addition of granulated biochars compared to unprocessed biochars (Liao et al. 2022b). The applications of granulated biochars to the green roof modules with *Sedum album* best reduced biochar erosion, showing no detectable biochar loss over the course of the 2-year experiment.

In contrast to the low erosion losses of granulated biochar, an average of 94% of the unprocessed biochars were lost due to wind and water erosion after 2 years. This is almost certainly mostly due to the small particle size and low bulk density of the unprocessed biochars and the fragmentation of the friable unprocessed biochars during and after application. Unprocessed wood-feedstock biochars also show uneven surfaces and a high aspect ratio (Liao and Thomas 2019), which likely contributes to erosion transport by wind and surface runoff. Our average of 94% of biochar loss is higher than a published figure of 82% of pyrogenic carbon loss with rainfall simulation (Rumpel et al. 2009), consistent with the green roof modules being exposed to wind erosion and assessed over a longer timeframe.

Contrary to our hypothesis, the organic dust suppressant Entac™ showed no effects on mitigating the erosion of unprocessed biochars. Although Entac™ can bind fine biochar particles to decrease the particle dispersal after application (Jin et al. 2019), the impervious surfaces created by the Entac™ may increase substrate surface runoff volume and runoff rate (Singh et al. 2003); thus, some biochar  may be lost with surface runoff. In addition, Entac™ can act to inhibit germination and early establishment of herbaceous plants (Kuttner and Thomas 2017), such as the native plants used in this study, and this may further diminish Entac™ effectiveness in biochar erosion control. As Entac™ degrades over time (Kuttner and Thomas 2017), the bonding between the biochar and substrate particles is likely to progressively decline and thus delay but not eliminate biochar erosion losses. The negligible effects of Entac™ on biochar erosion control contradicts the findings from prior studies, which reported > 90% wind erosion resistance for coal (Jin et al. 2019, 2022) and > 85% of water erosion resistance for dusts (Wang et al. 2021); however, prior studies involved assessments several days after applications of chemical dust suppressants to relatively even surfaces. The low particle erosion resistance with the Entac™ addition in the present study may be attributed to the longer duration of wind and water exposure and uneven surface of green roof substrates. Additional factors include high temperature fluctuations in the field, which may accelerate Entac™ degradation and/or decrease Entac™ binding strength (Li et al. 2020), and also the distinct properties of alternative dust suppressants (Zhang et al. 2018).

### Biochar granulation reduces total substrate erosion

In addition to reduced erosion losses of biochar, additions of granulated biochars substantially reduced total substrate erosion of green roof modules. This can be explained by several mechanisms. First, the substrates with the addition of granulated biochars presented the highest moisture content, which can reduce total substrate erosion by increasing particle cohesion and total mass. This is consistent with the observed negative correlation between substrate moisture and total substrate erosion (Fig. 3b). Second, the granulated biochar-amended substrates had higher bulk density than the unprocessed biochar-amended substrates and the control. Third, the amendment of granulated biochars can decrease substrate total porosity (Liao et al. 2022a) and thus may reduce the potential for particle transport and losses with discharge water. In addition, we suspect that the surface applications of the granulated biochars may reduce the exposure of fine substrate particles, thereby alleviating particle wind and splash erosion. In the long term, biochar additions may also enhance substrate aggregation (Blanco-Canqui 2017). Although unprocessed biochars and BCEntac showed no impacts on substrate moisture content at the end of the experiment, this is possibly due to the high biochar erosion loss. Biochar additions generally improve substrate water retention (Razzaghi et al. 2020), and this has specifically been shown with green roof substrates (Cao et al. 2014; Liao et al. 2022a). Thus, increased water retention early in the experiment likely contributed to reduced total substrate erosion in all biochar addition treatments. Decreased substrate erosion with biochar applications has been observed in agricultural studies (Blanco-Canqui 2017; Li et al. 2019).

Green roof modules showed a characteristic spatial pattern of substrate erosion, with higher erosion near the center of the modules; this pattern was most pronounced in unprocessed biochars and BCEntac treatments (Fig. 5). With low vegetation cover, the edges of the modules likely act as buffers to decrease the wind speed for the substrates behind the edges and also to restrict the substrate particles to deposit along the edges opposite to the wind directions. In contrast, substrates near the center of the modules had no surrounding objects to reduce wind speed, and thus particles were more directly exposed and eroded to the edges of the modules or completely lost with wind. Additionally, as the modules with the unprocessed biochars and BCEntac showed more pronounced edge-to-center differences than those with the granulated biochars and the control (Additional file 1: Fig. S3), we suspect that the erosion spatial distribution may be attributed in part directly to transportation of unprocessed biochar particles.

### *Sedum album* better reduces biochar and total substrate erosion than native plants

Green roof modules with *Sedum album* better reduced both biochar and total substrate erosion relative to those with the native plant mix. These distinct effects are likely due to differences in vegetation continuity and cover, plant water-use efficiency, and substrate moisture content. *Sedum album* spreads rhizomatously and characteristically forms a mat that reduces wind exposure. In addition, *Sedum album* generally shows more rapid and persistent early establishment of vegetation cover relative to native herbaceous plants on green roofs (Monterusso et al. 2005), which likely reduced erosion early in the experiment. *Sedum album* also shows facultative crassulacean acid metabolism (CAM) photosynthesis (Bachereau et al. 1998), and even among *Sedum* species shows low daytime transpiration, which thus minimizes substrate water loss (Wolf and Lundholm 2008; Starry et al. 2014). In the present study we observed improved substrate moisture content with *Sedum album* relative to the native plant mix, as well as enhanced biochar and substrate stability. In addition to the effects of high vegetation cover and low transpiration, the increased substrate moisture content in the *Sedum album* modules was possibly increased by maintenance of substrate depth (Fig. 2b). The negative relationships between vegetation cover and biochar and total substrate erosion emphasize the general importance of promoting vegetation cover in reducing biochar and total substrate erosion, particularly at early vegetation establishment stages.

In the present study biochars were applied as a topdressing, which is a likely practice for existing green roofs, and in many systems where repeated biochar amendment is required to enhance substrate properties and/or mechanical biochar incorporation is difficult (Verheijen et al. 2010). The coarse-textured FLL substrate used ensured that biochar particles were mixed to some extent with the substrate; however, surface application of biochar will generally increase erosion risks. Future studies should consider deep-band incorporation or pre-mixing of biochars and substrates prior to green roof installation. In addition, the results from the present study examine total biochar and substrate losses due to wind and water erosion. Since wind and water erosion have distinct processes and mechanisms, separate evaluation of wind and water erosion may be important to improve green roof configurations and model biochar and substrate erosion rates. In addition, while the present study found a distinct advantage of *Sedum* species compared to native species in reducing erosion losses, further exploration of appropriate regional native species for green roof use is a priority, since mixtures of native species have additional ecological benefits in terms of increasing biodiversity and resilience (Oberndorfer et al. 2007; Cook-Patton and Bauerle 2012). We also recommend future research on combinations of *Sedum* species and native plants to examine optimal plant combinations that are erosion resistant while providing a range of benefits on green roofs.

## Conclusion

Green roof systems are highly exposed and subject to erosion risks. We found that addition of granulated biochars strongly reduced biochar erosion and total substrate erosion over a 2-year period. Granulation of biochar increases biochar bulk density and particle size while retaining biochar’s benefits to substrate moisture-holding capacity. Our results thus suggest significant erosion control benefits of granulated biochars in green roof applications and provide a clear incentive for future research to optimize use of granulated biochars on operational green roofs.

## Supplementary Information

**Additional file 1.** **Table S1.** Plant species for native plant mix treatment. **Fig. S1.** Monthly total precipitation and mean speed of maximum wind gust during the experimental period (July 2019–October 2021). **Fig. S2.** Measurement locations based on the systematic sampling method. **Fig. S3.** Substrate edge-to-center difference in green roof modules.

## Data Availability

All data generated or analyzed during this study are included in this published article and its Additional files.
