# Supplementary material for: Biochar granulation reduces substrate erosion on green roofs
Source: Biochar. 2022 Oct 27;4(1):61. doi: 10.1007/s42773-022-00186-7 (PMC9613583; doi:10.1007/s42773-022-00186-7)
Supplement: Supplementary file 1 — Additional file 1. Table S1. Plant species for native plant mix treatment. Fig. S1. Monthly total precipitation and mean speed of maximum wind gust during the experimental period (July 2019–October 2021). Fig. S2. Measurement locations based on the systematic sampling method. Fig. S3. Substrate edge-to-center difference in green roof modules. [file 42773_2022_186_MOESM1_ESM.docx]

**Additional file 1 for:**

**Biochar granulation reduces substrate erosion on green roofs**

Wenxi Liao^a^ *, Melanie Sifton^a^, Sean C. Thomas^a^

^a^ Institute of Forestry and Conservation, John H Daniels Faculty of Architecture Landscape and Design, University of Toronto, 33 Willcocks St., Toronto, ON, M5S 3B3, Canada

* Corresponding author: Wenxi Liao

E-mail address: [wenxi.liao@mail.utoronto.ca](mailto:wenxi.liao@mail.utoronto.ca)

**Additional table:**

**Table S1.** Plant species and number of seeds or seedling for each green roof module with the native plant mix treatment.

| **Plant species** | **Number of seeds per module** | **Number of seedlings per module** |
| --- | --- | --- |
| **Direct seeding: July 2019** | | |
| *Rudbeckia hirta* (black-eye susan) | 30 |  |
| *Coreopsis lanceolata* (lanceleaf coreopsis) | 50 |  |
| *Asclepias tuberosa* (butterfly weed) | 9 |  |
| *Helenium autumnale* (sneezeweed) | 50 |  |
| *Lupinus perennis* (wild lupine) | 5 |  |
| *Geum triflorum* (prairie smoke) | 5 |  |
| *Dalea purpurea* (purple prairie clover) | 100 |  |
| *Campanula rotundifolia* (harebell) | 20 |  |
| **Transplantation: September 2020** | | |
| *Agastache foeniculum* (anise hyssop) |  | 5 |
| *Achillea millefolium* (yarrow) |  | 5 |
| *Gaillardia aristata* (great blanket flower) |  | 5 |

**Additional figures:**

**Fig. S1.** Monthly total precipitation and mean speed of maximum wind gust during the experimental period (July 2019 – October 2021). Blue bars and the orange line indicate total precipitation and average speed of maximum wind gust in each month, respectively.

**Fig. S2.** Measurement locations based on the systematic sampling method. X-axis and y-axis indicate the length and width of the green roof module.

**Fig. S3.** Substrate edge-to-center difference in green roof modules. Bars plot mean substrate edge-to-center difference ± 1 standard error (n = 3 replicates per treatment).
